# Socioeconomic and Environmental Factors Associated with Child Undernutrition and Growth Failure in Eastern Africa

**DOI:** 10.3390/nu18040607

**Published:** 2026-02-12

**Authors:** Maryam Siddiqa, Gulzar Shah, Tahreem Asif, Asifa Kamal, Bushra Shah

**Affiliations:** 1 Department of Mathematics & Statistics, International Islamic University, Islamabad 44000, Pakistan; maryam.siddiqa@iiu.edu.pk (M.S.); tmiasif4@gmail.com (T.A.); 2 Department of Health Policy and Community Health, Jiann-Ping Hsu College of Public Health, Georgia Southern University, Statesboro, GA 30460, USA; bs06779@georgiasouthern.edu; 3 Department of Statistics, Lahore College for Women University, Lahore 54000, Pakistan; asifa.kamal@lcwu.edu.pk

**Keywords:** Composite Index of Anthropometric Failure (CIAF), Eastern Africa, child undernutrition, growth failure, social determinants of health, maternal education, nutrition equity, food and water security, health disparities

## Abstract

**Background and Objective:** This study examines the factors associated with child undernutrition among children under five in Ethiopia, Kenya, Madagascar, and Tanzania. It uses the Composite Index of Anthropometric Failure to measure the full burden of undernutrition, combining weight-for-height (WHZ), weight-for-age (WAZ), and height-for-age (HAZ) indicators. This approach captures children facing multiple forms of failure that single indicators miss. **Methods:** The study analyzed 37,570 children using nationally representative Demographic and Health Survey (DHS) data for Ethiopia 2019, Kenya 2022, Madagascar 2021, and Tanzania 2022. A binary logistic regression model identified key predictors of child undernutrition across countries. **Results:** The prevalence of anthropometric failure ranged from 24% to 44%. Higher parental education, child’s age, socioeconomic status, child’s sex, and a postnatal checkup within 2 months were associated with a lower odds of anthropometric failure. Children of educated mothers in Ethiopia (AOR = 0.547) and Tanzania (AOR = 0.606) had better outcomes. Educated fathers in Kenya (AOR = 0.589) and Madagascar (AOR = 0.369) reduced the risk of child undernutrition. Children aged 13–24 months had a higher risk in all countries. In Madagascar (AOR = 0.309), children who received a postnatal checkup had a decreased risk of malnutrition. Children from rich households in Ethiopia (AOR = 0.645) and from middle (AOR = 0.683) and rich (AOR = 0.535) households in Kenya had significantly lower odds of undernutrition. In comparison, female children had lower odds of anthropometric failure in all four countries. **Conclusions:** Viewed through a nutrition equity lens, these findings underscore the importance of recognizing how the intersectionality of anthropometric failures disproportionately affects children from poorer households and communities with limited access to education and postnatal care. This study advances existing knowledge by using the Composite Index of Anthropometric Failure to show overlapping and hidden forms of undernutrition. The findings identify child age, parental education, postnatal checkup, child sex, and socioeconomic status as shared priorities for reducing undernutrition. The results provide country-specific insights for designing integrated, evidence-based nutrition interventions in Eastern Africa.

## 1. Introduction

Undernutrition is the leading cause of death for children aged five and under in developing countries, contributing to multifaceted hardship for individuals and communities [1]. Key factors for optimal child nutrition include access to healthcare services, age-appropriate feeding practices, and adequate nutrition [2].

Childhood malnutrition triggers a chain reaction of challenges with long-lasting detrimental effects influenced by factors such as inadequate feeding practices, poor healthcare access, and maternal malnutrition [3]. The Global Nutrition Report 2021 reported that 35.5% of children are stunted, 19.3% are wasted, and 7.7% are severely wasted globally [4]. In Africa, the prevalences of stunting and wasting were 34.8% and 6.8%, respectively [5], highlighting the undesirable status of child malnutrition [6].

The global prevalence of stunting among children aged 5 years and under has declined from 41.5% in 2000 to 30.7% in 2020. Africa is the only continent where the overall number of stunted children has increased, growing from 54.4 million in 2000 to 61.4 million in 2020 [7]. The Food and Agriculture Organization reported that 58.9% of African children had moderate to severe food insecurity [8]. Studies show that maternal education and other socioeconomic measures are critical determinants of child malnutrition in less privileged countries [1,9]. The most critical factors affecting child malnutrition reported are gender, birth size, maternal education, maternal body mass index, socioeconomic status, and maternal autonomy [10,11,12]. Furthermore, some studies have reported that parental education, maternal body mass index (BMI), rural residence, economic status of the household, and type of residence are significantly associated with child malnutrition [13,14,15]. Childhood malnutrition is shaped by a complex interplay of biological, behavioral, household, and community factors, such as inadequate and poorly varied diets, infections, and insufficient maternal nutrition [16,17,18,19]. Studies have outlined that child malnutrition is also linked with household socio-economic status, gender discrimination, maternal education, and health-care access [20,21]

Many existing African studies of malnutrition have estimated nutritional status through conventional anthropometric measures such as stunting, wasting, and underweight. However, these traditional measures do not provide an all-inclusive measure of undernutrition in the population and can mask the actual undernutrition status due to overlap [22]. Therefore, the Composite Index of Anthropometric Failure (CIAF), an alternative single indicator of undernutrition, can more accurately assess nutritional status and identify vulnerable populations [23]. Few studies in various African countries have recently used this unconventional measure of undernutrition [24,25,26,27,28,29]. None of these studies has comprehensively addressed child, maternal, and environmental factors in nationwide analyses of child malnutrition [26,27,28].

The CIAF combines stunting, wasting, and underweight into a single measure, overcoming the limitations of traditional single indicators. Researchers use CIAF to assess the full extent of malnutrition, particularly in low- and middle-income countries, where single indicators often miss concurrent failures [30,31]. CIAF helps determine the true scale of anthropometric failure in low-resource settings, enabling targeted interventions in these countries and helping researchers identify a combination of environmental, maternal, and socioeconomic factors that remain hidden when using separate measures [32]. It is suitable for multi-country DHS analyses and trend studies, enabling consistent comparisons across nations and survey years [33,34]. The index supports focused interventions through subgroup analysis, improving policy and resource distribution [35]. This analysis differs from others by examining child, maternal, environmental, and socio-demographic factors across four countries using a unified method. Traditional indicators overlap, potentially underestimating the total burden. CIAF identifies children with multiple concurrent failures, the group most at risk of illness and death, who often remain unseen in programs addressing single conditions. It defines six categories of failure, thereby helping policymakers prioritize the most severely affected groups for immediate intervention.

This study uses CIAF with DHS data from four Eastern African countries to reveal a more comprehensive estimate of overall child undernutrition by capturing overlapping failures, offering stronger evidence for integrated nutrition planning and monitoring. It aimed to address gaps in the literature by utilizing the CIAF to determine the prevalence of undernutrition in Eastern African children aged five years and under, specifically in Tanzania, Kenya, Madagascar, and Ethiopia, while considering contributing factors from maternal, social, and child-level determinants, using the latest available nationwide DHS datasets of Ethiopia (EDHS-2019), Kenya (KDHS-2022), Madagascar (MDHS-2021) and Tanzania (TDHS-2022). These countries were selected based on three criteria. First, they are part of Eastern Africa and share a regional burden of child undernutrition. Second, recent and complete DHS datasets were available for all four countries, ensuring methodological consistency and data comparability. Third, they represent diverse socioeconomic and environmental contexts, which allow examination of both common and distinct factors influencing child undernutrition. Beyond single-country DHS analyses and studies focused on stunting, wasting, or underweight, this study uses CIAF to quantify overlapping anthropometric failures. It applies a consistent, survey-adjusted modeling approach across four countries. This cross-country design enables identification of shared determinants and country-specific patterns that are not visible in isolated national analyses.

## 2. Materials and Methods

### 2.1. Dataset and Study Population

This study was based on the analysis of the most recent available DHS data sets for Ethiopia (EDHS-2019; ETKR81SV data file), Kenya (KDHS-2022; KEKR8CSV data file), Madagascar (MDHS-2021; MDKR81SV data file), and Tanzania (TDHS-2022; TZKR82SV data file). The study population comprises children with complete and accurate anthropometric measurements aged 0–60 months. DHS data quality requirements were followed in the study of children whose anthropometric z-scores were flagged or missing. In particular, z-scores for height-for-age (HAZ), weight-for-height (WHZ), and weight-for-age (WAZ) that fell outside the biologically reasonable limits established by the DHS were deemed invalid and excluded from analysis. The Demographic and Health Surveys (DHS) conduct nationally representative studies, using a two-stage stratified sampling design. Stratification for these four DHSs was conducted by rural-urban status. A total of 11,516 primary sampling units (PSUs) were selected across the four countries, including 7981 from rural areas (6360 + 1026 + 418 + 177) and 3989 from urban areas (2790 + 666 + 211 + 322). In each selected cluster, a complete list of households was obtained, and qualifying households were selected for interviews. In this study, 95,085 married women and men from various regions of these four countries were surveyed regarding the level of child malnutrition. To preserve sample size and maintain comparability across countries, missing covariate values were handled using mode imputation only for categorical variables with low levels of missingness. Children with missing anthropometric measurements that could not be classified were excluded. Mode imputation reduces listwise deletion, helping retain statistical power and limit distortion of country-specific estimates [36]. No imputation was applied to the CIAF outcome variable. Sensitivity analyses comparing the primary model with complete-case models showed no meaningful changes in direction or magnitude of associations.

Data from approximately 37,570 children aged five and under 5 years of age were used (4825 from Ethiopia, 20,319 from Kenya, 5744 from Tanzania, and 6682 from Madagascar) to investigate the determinants of malnutrition.

### 2.2. Study Variables and Measurements

The CIAF was applied to estimate the overall burden of child undernutrition, capturing overlapping anthropometric failures not reflected in individual indicators, following the standard classification framework originally proposed by Svedberg [37] and later operationalized by Nandy et al. [38]. CIAF combines three conventional anthropometric indicators, height for age, weight for height, and weight for age, each defined using a z-score threshold below minus two standard deviations from the WHO reference population. Based on the presence and overlap of these indicators, children were classified into seven mutually exclusive groups. Group A comprises children without anthropometric failure. Group B includes wasting only. Group C includes wasting and underweight. Group D includes wasting, stunting, and underweight. Group E includes stunting and underweight. Group F includes stunting only. Group Y includes only the underweight. Children in groups B–Y were considered undernourished; group A served as the reference. For regression analysis, CIAF was operationalized as a binary variable, where CIAF equals one indicates the presence of any anthropometric failure and CIAF equals zero indicates no failure. No imputation was applied to the CIAF outcome variable. The detailed summary statistics of all potential independent variables are presented in Table 1.

### 2.3. Statistical Analysis

In the descriptive analysis, sample characteristics of maternal, child, and socio-demographic factors are provided. The associated risk variables for undernutrition were evaluated using binary logistic regression, with CIAF as a measure of anthropometric failure and child undernutrition in children aged 5 years and under. The Adjusted Odds Ratio (AOR), defined as the ratio of the exposed group’s probability of the outcome to the unexposed group’s probability of the outcome, was used to quantify the strength of association. A backward stepwise elimination strategy was used to select risk factors for the final model. An iterative variable-selection process known as “stepwise backward elimination” eliminates irrelevant variables one at a time, beginning with the entire model. All variables were incorporated into the model at the first stage of fitting the multivariable model. Subsequently, the variable with the largest *p*-value or smallest F-statistic was removed at each stage, and the model-fitting procedure was repeated until only the components substantially associated with the study outcome were selected and retained. The sample weights were used in all the analyses. The weights were obtained from the DHS variable V005 and scaled by 1,000,000 to create the sampling weights. The weights account for the sampling design and provide nationally representative estimates. The clustering and stratification were accounted for by specifying the primary sampling unit (V021) and the stratification variable (V022) in the survey commands across all analyses, using the appropriate survey design settings (svyset) in Stata. All analyses accounted for DHS sampling weights, clustering, and stratification using survey-adjusted statistical procedures in Stata (Version 13; StataCorp LLC, College Station, TX, USA) [39] and SPSS (Version 20; IBM Corp., Armonk, NY, USA), in accordance with DHS analytical guidelines.

In this study, binary logistic regression was used to identify independent determinants of anthropometric failure within a predefined CIAF-guided framework. Backward stepwise regression was used to obtain a parsimonious model while retaining variables with independent associations after adjustment. This approach is appropriate for analyses involving associated socioeconomic and demographic variables, where full models risk overfitting and unstable estimates. All candidate variables were included in the initial model based on prior evidence and theoretical relevance to the child, parental, household, and environmental domains. Variables were removed sequentially using a consistent significance threshold, while key framework variables were retained regardless of statistical strength. The stepwise procedure was then applied to obtain a parsimonious model and reduce potential multicollinearity and over-fitting among predictors. This strategy supported model stability and improved the interpretability of adjusted associations. To address concerns about instability and inflated significance, variance inflation factors were examined, and no evidence of multicollinearity was found. Literature explicitly reported that the use of backward stepwise or backward elimination procedures is appropriate for multivariable model development and identification of independent predictors of child undernutrition after inclusion of all conceptually relevant variables in the model [40,41,42]. These references support both approaches as established practices in recent large-scale, survey-based nutrition research.

## 3. Results

Table 1 summarizes the descriptive statistics of risk factors associated with child undernutrition. This current study included a total of 37,570 children. Most of the children lived in rural regions: 76.92% in Ethiopia, 65.77% in Kenya, 80.90% in Madagascar, and 72.75% in Tanzania. The majority of households in all countries used unimproved toilet facilities: Ethiopia (79.77%), Kenya (57.78%), Madagascar (75.22%), and Tanzania (67.67%). The different socio-economic categories were equally represented in the samples from Ethiopia and Madagascar (50%) and from Kenya and Tanzania (almost 41%). A minority of children in Kenya (34%) and Madagascar (42%) had educated fathers, whereas more than half of the children in Tanzania (56%) did. The majority of families used an unimproved water source: approximately 81% in Ethiopia, Madagascar, and Tanzania, and around 70% in Kenya. Approximately half of the children (51%) were boys. Approximately 50% of children across the four countries were in the 2nd to 4th birth order. The majority of children in Ethiopia (86.60%), Kenya (74.03%), and Madagascar (60.81%) did not receive a baby postnatal checkup; however, in Tanzania, 66.48% did. More than half of children in Ethiopia (66.29%), Kenya (52.80%), Madagascar (57.65%), and Tanzania (52.16%) were breastfed, and the majority of children, almost 80% in all countries, were initiated into breastfeeding immediately. In Ethiopia, 54% of children had mothers with no formal education, whereas in Kenya, Madagascar, and Tanzania, approximately 35%, 43%, and 52% had mothers at least at the primary level. A large majority of mothers were working in Madagascar (86.71%), Tanzania (66.23%), and Kenya (50.51%). A majority had an iron intake during pregnancy (57.98%). Women mainly were non-smokers: Ethiopia (89.63%), Kenya (72.67%), Madagascar (82.23%), and Tanzania (99%).

Table 2 presents the construction of the response variable (CIAF) using traditional measures of stunting, wasting, and underweight. We used CIAF to categorize the overall frequency of anthropometric failure into at least one of the six groups (Groups B–Y) that demonstrated failure. The CIAF indicates that about 57.75% of Ethiopian, 75.58% of Kenyan, 55.57% of Madagascar, and 67.73% of Tanzanian children were not undernourished (Group A). The most prevalent undernourished category was Group-F 868 (16.44%) in Ethiopia,1677 (9.42%) in Kenya,1142 (19.26%) in Madagascar, 323 (7.98%) in Tanzania. Kenya 267 (1.50%) in Group-Y and Tanzania 3 (1.60%) had the lowest prevalence of Group D among anthropometric failure groups.

A single measure of anthropometric failure (CIAF) from seven groups of children (groups A–Y) by excluding children not in anthropometric failure (group A) and including all children who were any type of undernourished with their combinations (groups B–Y) is presented in Table 3.

Table 3 shows that the percentage of children aged 5 and under who suffered undernutrition (1–26% in all four countries) was higher for children of uneducated mothers than for those of educated mothers. The percentages of children in Madagascar (39.16%) and Tanzania (21.40%) experiencing undernutrition were higher among children of employed mothers. Conversely, in Kenya (13.48%), children of non-working mothers experienced higher rates of undernutrition. The frequency of malnourished children varies by age group, with the highest rates seen in Madagascar (10.24%) in the age group 13–24 and Ethiopia (9.83%) in the age group 25–36. The prevalence of undernutrition in male children was (22.75%) in Ethiopia, Kenya (13.54%), Madagascar (24.22%), and in Tanzania (17.77%), and remained consistently higher for male children as compared to girls in all four countries. Compared with girls, boys bear a greater burden of undernutrition among all groups. A majority of children aged five years and under in all four countries were malnourished, which included Kenya (16.43%), Ethiopia (25.17%), Madagascar (23.61%), and Tanzania (16.14%). Undernutrition rates were higher in children from low-income households compared with high-income households. The percentage of malnourished children living in rural areas was approximately 35–36% in Ethiopia and Madagascar, alongside 18–25% in Kenya and Tanzania. Children aged five and under who lived in rural areas had a greater chance of undernutrition.

Table 4 supports a multilevel analytical framework linking child, maternal, household, and environmental factors to undernutrition measured using the CIAF. At the child level, age and sex showed the most consistent associations across all countries. Female children had lower odds of anthropometric failure in Ethiopia, Kenya, Madagascar, and Tanzania. This pattern suggests biological and care-related differences operating early in life. Child age showed a strong and consistent gradient. Children aged 7 to 12 months and 13 to 24 months had higher odds of anthropometric failure compared with those aged 0 to 6 months. The peak risk observed among children aged 13 to 24 months reflects the period of complementary feeding and increased exposure to infection, reinforcing the central role of early life nutritional transitions in the CIAF framework. Other child level factors, including birth order, birth size, milk consumption, breastfeeding initiation timing, and postnatal checkup, showed limited or country-specific associations, indicating weaker or context dependent pathways.

At the maternal level, education emerged as an important determinant in selected settings. In Ethiopia, secondary and higher maternal education showed strong protective associations with anthropometric failure. In Tanzania, primary and secondary education also showed reduced odds. These findings support a pathway where maternal education influences child nutrition through knowledge, caregiving practices, and health service use. Maternal smoking, iron intake during pregnancy, breastfeeding history, and maternal occupation showed no significant associations, suggesting limited direct effects within the adjusted model or indirect effects mediated through other factors.

At the household level, socioeconomic status showed a clear association in Ethiopia and Kenya. Children from rich households had lower odds of anthropometric failure compared with those from poor households. In Kenya, children from middle households also showed reduced odds. These results align with a resource-based pathway where household wealth influences food access, healthcare utilization, and living conditions, which collectively shape CIAF outcomes. The absence of significant associations in Madagascar and Tanzania indicates contextual variation in how household resources translate into nutritional status.

At the environmental level, place of residence, drinking water source, and sanitation facility showed no statistically significant associations after adjustment. These findings suggest that their effects on undernutrition operate indirectly through household and maternal pathways rather than as independent predictors within the multivariable model.

Table 5 indicates the adequacy of the fitted model of CIAF for all four countries models. Hosmer–Lemeshow and Pearson Chi-squared tests showed that fitted models of undernutrition based on CIAF were insignificant at *p* > 0.05, indicating that all four models were well fitted.

**Figure 1:**
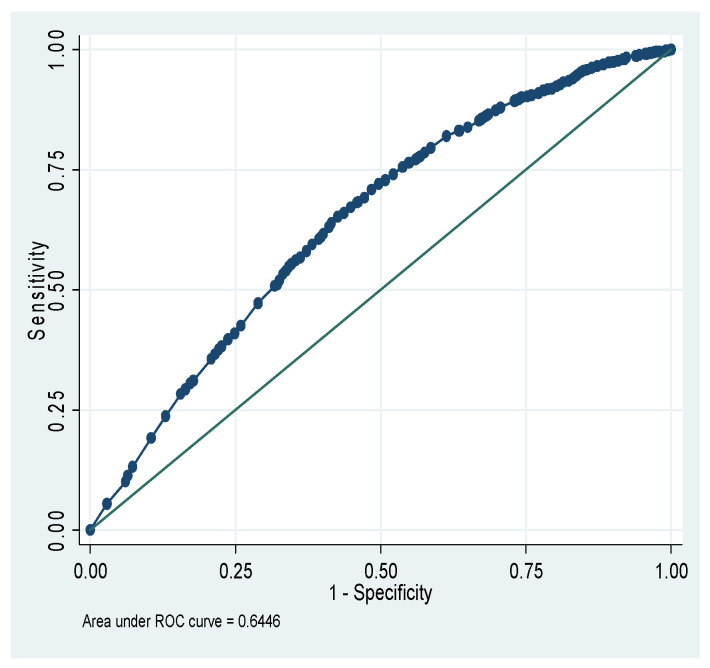
Receiver Operating Curves (ROC) of the Composite Index of Anthropometric Failure model for Ethiopia.

**Figure 2:**
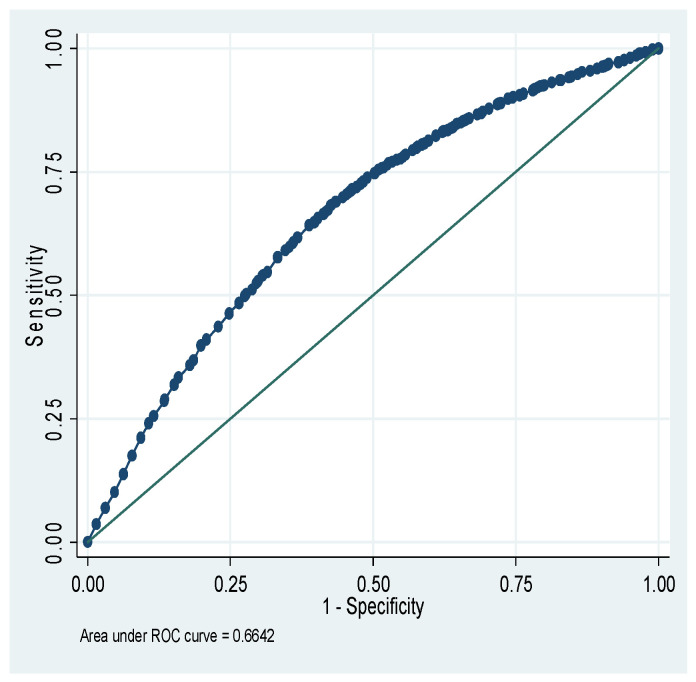
Receiver Operating Curves (ROC) of the Composite Index of Anthropometric Failure model for Kenya.

**Figure 3:**
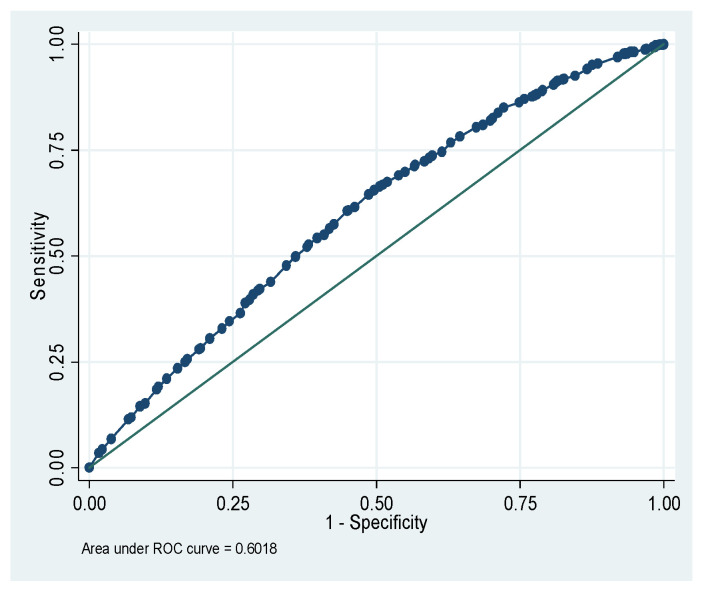
Receiver Operating Curves (ROC) of the Composite Index of Anthropometric Failure model for Madagascar.

**Figure 4:**
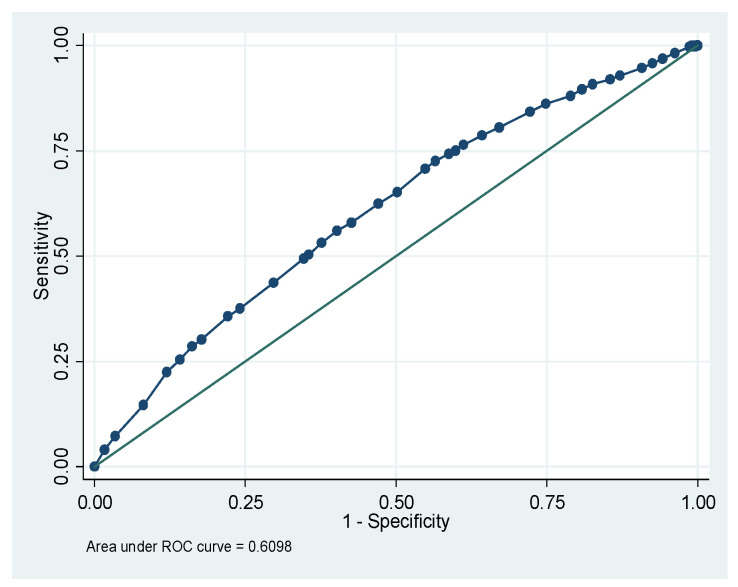
Receiver Operating Curves (ROC) of the Composite Index of Anthropometric Failure model for Tanzania.

Figure 1 shows that the total area under the receiver operating characteristic (ROC) curve is 64.46% for the CIAF model of Ethiopia. Figure 2 shows that the total area under the ROC is 66.42% for the CIAF model of Kenya. Figure 3 shows that the total area under the ROC is 60.18% for the CIAF model of Madagascar. Figure 4 shows that the total area under the ROC is 60.98% for the CIAF model of Tanzania. Moreover, all four ROCs lie in the upper-left quadrant, indicating better performance for each model.

## 4. Discussion

This study uses the CIAF to assess child undernutrition in four Eastern African countries. The analysis combines large DHS datasets in a single framework to give clear, comparable results. Most of the earlier studies focused on stunting, wasting, or underweight as separate measures. This study looks at all forms together to show the full scale of nutritional deprivation among children under five. The findings reveal a larger share of children facing more than one type of failure, showing that single indicators can miss part of the problem. The results also highlight patterns of inequality across households and communities. Using the Composite Index provides stronger evidence for programs that address multiple forms of under nutrition simultaneously. It helps policymakers identify high-risk groups and design integrated actions. The approach supports better tracking of progress toward national and regional nutrition goals. Child undernutrition is a serious public health concern in Eastern African countries. This study investigated characteristics associated with undernutrition prediction in African children aged 5 and under and examined undernutrition levels in children using data from Ethiopia (EDHS-2019), Kenya (KDHS-2022), Madagascar (MDHS-2021), and Tanzania (TDHS-2022). The CIAF was used to determine the prevalence of undernutrition, providing insight into the complex nature of child undernutrition. The CIAF index, which uses different types of anthropometric failure, showed concerning higher rates of child undernutrition for Ethiopia, Kenya, Madagascar, and Tanzania in our results. The CIAF can identify children with multiple anthropometric failures and provides a single measure of nutritional status. Few multivariable modeling studies, particularly in Ethiopia, Kenya, Madagascar, and Tanzania, have considered CIAF as an indicator of undernutrition.

The analysis follows a CIAF-guided analytical model linking child level, parental, household, and environmental determinants of under nutrition. The model assumes distal environmental conditions shape household resources. Household and parental characteristics influence caregiving capacity. Child characteristics reflect biological vulnerability during early growth. Overall, results highlight socioeconomic status, parental education, child sex, early childhood age, and maternal education factors as key determinants of anthropometric failure, with notable variation across countries.

In the current study, the prevalence of under nutrition among children aged 5 and under is 42.25% in Ethiopia, 24.42% in Kenya, 44.43% in Madagascar, and 32.27% in Tanzania, as determined by the CIAF, which is a cause for concern. Findings exposed the following factors significantly associated with under nutrition in univariate analysis: place of residence, source of drinking water, toilet facility, socioeconomic status, milk consumption, initiation of breastfeed, birth order number, sex of child, size of child at birth, child’s age in months, breastfed ever, intake of iron during pregnancy, mother education, and mother occupation (*p*-values < 0.05). Afterward, in binary logistic analysis, factors associated with under nutrition are: socio-economic status in Ethiopia and Kenya, father’s education in Kenya and Madagascar, sex of child in all countries, baby postnatal checkup in Madagascar child’s age in months in all countries, mother’s education in Ethiopia and Tanzania with *p*-values < 0.05.

Malnutrition was shown to be much less common among children from higher socioeconomic backgrounds and mothers with higher levels of education than in children from lower socioeconomic backgrounds. Higher socio-economic often means better access to variety and nutritious food, stable household income to support adequate diet, safer living surroundings, and more possibilities for preventative healthcare and growth monitoring all of which minimize the risk of chronic and acute undernutrition [43]. According to a study [44] evaluating child undernutrition in various contexts children from middle-class and upper-class households were substantially less likely to be malnourished. The results support the idea that higher incomes and easier access to wholesome food and medical treatment lower the risk of undernutrition.

According to a study, male children had greater prevalence rates of underweight, stunting, and wasting than female children, indicating that boys were more impacted by malnutrition in that region [45]. Subsequently addressing both socioeconomic and environmental factors, extensive varied data reveals that girls frequently have lower rates of stunting, wasting, and underweight than boys [46]. At certain years of age, boys may participate in more physically demanding play and activity patterns, raising energy needs that aren’t satisfied in situations with restricted resources though this can frequently be related to broader societal issues [47]

According to a study [48], postnatal visits by a medical professional within 48 h of delivery were substantially associated with improved child feeding outcomes, including dietary diversity and appropriate food introduction, which are important determinants of nutritional status and risk of malnutrition in infancy and early childhood. Another study [49] shows that specifically measured anthropometric nutritional deficiency outcomes, merely to baby postnatal checkups, interventions incorporated into postnatal care, such as nutritional counseling, breastfeeding support, and caregiver education, have been demonstrated to improve early growth outcomes and feeding practices, which contribute to reductions in undernutrition.

Child age was consistently associated with CIAF across all countries. Children aged 13–24 months had higher odds of anthropometric failure than younger children (0–12 months), indicating that with increasing age, the risk of undernutrition rises. Based on studies [50], children between the ages of 13 and 24 months had substantially higher odds of malnutrition than younger infants. This suggests that the risk of malnutrition increases dramatically as age grows within the 6–24-month age range. This is frequently caused by rapid growth, which increases nutrient requirements and reduces the benefits of exclusive nursing. A child’s nutritional needs increase rapidly after exclusive breastfeeding ends, typically around six months. Inadequate supplemental feeding in terms of quantity, variety, or frequency results in inadequate nutritional intake, which can cause stunted growth, weight loss, or deficiency that raises the risk of infection [51].

The estimates were not statistically significant and were directionally similar across settings. This consistency strengthens confidence in the observed association, though the cross-sectional nature of the data limits inference about growth trajectories over time. Maternal undernutrition can be a significant reason because malnourished mothers may not feed sufficient breast milk or may have inadequate nutrient supplies to support fetal growth during pregnancy. Although previous DHS-based studies often report increasing undernutrition after infancy, our analysis found higher CIAF prevalence among older children than among those under 6 months. Several factors may explain this finding. Since CIAF measures current anthropometric status, some children may have recovered from earlier growth faltering. This result suggests the need for further age-specific analyses of nutritional recovery and mortality patterns to understand CIAF dynamics across childhood better.

Anthropometric failure among children is inversely correlated with maternal education. The empirical findings indicated that children of secondary- or higher-educated mothers were less likely to experience undernutrition than those of less educated mothers [52,53,54]. This is because maternal education increases awareness of health information, which improves child-feeding practices and benefits children’s health. Studies [41] in underdeveloped countries have found that maternal education is the most widely recommended primary approach for enhancing child health [41], as it is also linked to the family’s socioeconomic status and children’s nutritional status. Future parents who receive formal education may be better equipped to identify illness, pursue appropriate treatment, accept contemporary medications, and understand medical advice for managing children’s conditions. Our results align with those of other studies [41], which revealed that children of educated fathers are less likely to experience undernutrition than children of uneducated fathers. Educated fathers are more likely to make well-informed decisions regarding dietary habits, preventive healthcare, and hygiene because they often have better access to health and nutrition information. This enhances the child’s environment to support overall nourishment [55].

The significance of socioeconomic and knowledge-based routes in influencing childhood nutrition is underscored by the finding that fathers’ education independently reduces undernutrition, even when mothers’ education remains a powerful predictor, particularly when both parents are educated [56]. This finding underscores the importance of fathers’ education for their children’s health. Parental factors showed consistent protective associations. Maternal education in Ethiopia and Tanzania paternal education in Kenya and Madagascar was associated with lower malnutrition risk. Education reflects knowledge of child feeding, health service use, and household decision making. These factors operate at the household level and influence daily care practices. Although the direction of association of maternal education was consistent with prior evidence, confidence intervals, particularly Ethiopia and Tanzania, were in normal range. This indicates limited precision and suggests heterogeneity within education categories. The results support an association between maternal education and child nutritional status rather than a definitive protective effect. Similarly, father education confidence intervals were also in normal range, indicating differences in sample distribution and subgroup size. These results should therefore be interpreted as context-specific associations.

Overall, the results align with a hierarchical framework in which child biological factors and early life stages exert the strongest direct influence on anthropometric failure, while maternal education and household socioeconomic status act as upstream determinants. Environmental factors appear mediated. This structure justifies the inclusion of variables across multiple levels and provides a coherent basis for interpreting CIAF-based undernutrition across diverse country contexts.

These patterns indicate sampling variability, small subgroup sizes, and contextual diversity. Point estimates for these variables should be interpreted with caution and not as precise measures of effect. Overall, the results support the CIAF-guided analytical framework by demonstrating associations across child, parental, household, and environmental domains interact to shape overall anthropometric failure. Composite measures such as the CIAF are particularly valuable in equity-oriented research because they capture multiple, overlapping forms of deprivation that are often concentrated among the most disadvantaged groups and may remain hidden when single indicators are used [57].

The analysis compared key factors linked to child undernutrition across Ethiopia, Kenya, Madagascar, and Tanzania using the CIAF. The results show both shared and country-specific patterns. In all four countries, older children faced a higher risk of undernutrition, showing that the first two years of life remain a critical window for nutrition action. Maternal education had a strong protective effect in Ethiopia and Tanzania, while paternal education mattered more in Kenya and Madagascar. This difference suggests that household decision-making roles shape how parental education influences child nutrition. Sex of child, postnatal checkup, and socio-economic status also influence nutritional status. These differences highlight how structural, economic, and social factors interact within each country. The results support country-specific policy approaches while reinforcing the need for regional strategies that target early childhood nutrition, parental education, and safe water access. The observed patterns of anthropometric failure, when viewed through a nutrition equity lens, highlight persistent social and structural inequities rather than isolated individual behaviors. Previous research evidence indicates that child undernutrition in low and middle-income countries is strongly shaped by parental education, household socioeconomic conditions, and access to health services. The synergistic effect of these disadvantages collectively determines children’s vulnerability to nutritional deprivation and their opportunities for recovery [58].

This study strengthens the literature by showing child undernutrition in Eastern Africa reflects more than food supply and parental behavior. Social determinants shape nutritional outcomes across settings. A health equity perspective grounded in regional cultural norms highlights structural and environmental inequities linked to intergenerational nutritional disadvantage. Action on these upstream determinants supports the reduction in downstream disparities associated with undernutrition. Practice-based and community-engaged educational interventions support sustained improvement in child nutritional status within low-resourced settings [46]. This study compares child undernutrition across Ethiopia, Kenya, Madagascar, and Tanzania using the CIAF. The results show clear differences in the scale of undernutrition between countries. In Ethiopia and Tanzania, maternal education strongly influenced child nutrition, reflecting the role of women’s knowledge. In Madagascar and Kenya, father education reduced the risk of undernutrition, underscoring the role of parental awareness in better feeding and care. Older children faced higher risks in all countries, showing the need for focused attention during early childhood. These country-specific differences reveal how social, economic, and environmental conditions shape child nutrition outcomes. The comparative view supports policies that strengthen parental education and target the first two years of life while promoting regional cooperation to reduce child undernutrition. This study advances existing knowledge by moving beyond single measures of child undernutrition. It uses the CIAF to capture all forms of nutritional deprivation in one measure. This approach identifies children who experience multiple types of failure, a pattern that traditional indicators overlook. The analysis compares four Eastern African countries using a consistent method and highlights differences in the combined burden of undernutrition. The results reveal new evidence on how multiple deprivations overlap among children in varied settings. This adds depth to the understanding of child nutrition patterns in the region. The findings support stronger and more targeted interventions that address the combined nature of undernutrition rather than treating each form separately.

Although this study followed a rigorous analytical approach, limitations inherent to cross-sectional DHS data should be acknowledged. The cross-sectional design precludes establishing temporal or causal relationships between exposures and child nutritional outcomes. Associations observed may reflect correlations rather than direct effects, as both predictor and outcome variables were measured at a single point in time. The findings identify associates of anthropometric failure rather than determinants. The reliance on self-reported information, particularly regarding household conditions and maternal characteristics, introduces the risk of recall and reporting bias. Moreover, variations in survey timing across the four countries (2019–2022) may contribute to subtle temporal inconsistencies, given potential changes in policy, economic conditions, or health interventions during those years. Although DHS data are nationally representative, some population subgroups, especially those in remote or marginalized settings, may remain underrepresented. This could limit the generalizability of the findings and underestimate the true burden of undernutrition in hard-to-reach populations. Finally, unmeasured confounding factors, such as regional food insecurity, household dietary diversity, micronutrient consumption or health service accessibility, may influence the associations observed and should be explored in future longitudinal or mixed-method studies. Lastly, the CIAF does not account for other aspects of undernutrition, such as vitamin deficits, even if it offers a thorough assessment of anthropometric failure. Despite these drawbacks, the application of sizable, nationally representative datasets and internationally accepted techniques offers solid insights that contribute to child undernutrition in Eastern Africa. Future longitudinal and multilevel analyses are required to assess directionality and stability of these associations across contexts.

This study adds new value by using the CIAF to provide a complete estimate of child undernutrition in Eastern Africa. Previous DHS-based studies in Africa treated stunting, wasting, and underweight as separate indicators, which underestimated the overall burden. By applying CIAF, the study captures overlapping forms of anthropometric failure and reveals the true scale of undernutrition. The work also uses the most recent DHS data from four Eastern African countries, providing a rare regional comparison in prior analyses. The results identify how parental education, child’s age, child’s sex, postnatal checkup, and socioeconomic status jointly influence the risk of undernutrition, providing clear evidence for integrated social and health interventions. The CIAF approach changes how malnutrition is assessed, shifting from a single-condition assessment to a comprehensive evaluation. This approach enables assessment of how associations among child, parental, household, and environmental factors differ across settings, using a consistent analytical framework. Together, these contributions provide a more comprehensive and comparable picture of patterns of child undernutrition in Eastern Africa than single-indicator or single-country studies. This perspective helps policymakers design coordinated interventions that target the combined effects of socioeconomic and environmental factors, improving the relevance of child nutrition programs in the region.

## 5. Conclusions

Across the four countries, approximately one in three children under five experienced at least one anthropometric failure as measured by CIAF. A CIAF-guided framework clarified how child characteristics, parental socioeconomic factors, household conditions, and environmental resources jointly shape the risk of undernutrition. Parental characteristics, including maternal and paternal, were consistently associated with lower rates of anthropometric failure, highlighting the links among caregiving capacity, household socioeconomic conditions, and children’s nutritional status. Child age further modified vulnerability during older growth periods. A clear analytical framework strengthens interpretation and supports equity-focused nutrition strategies. The findings support targeted, integrated interventions for children aged 7–24 months and for households with low parental education and limited socioeconomic resources. Programmatically, this includes strengthening support for infant and young child feeding, infection prevention, and early postnatal contact, alongside social protection strategies that improve household food security. Although water and sanitation variables were not independently associated in adjusted models, sustained investment remains essential as a foundational condition for child health. Coordinated action across health, education, and social protection sectors is needed to reduce overlapping anthropometric failure identified through CIAF. The CIAF-based findings, framed as equity issue, rooted in social and community conditions, support policies that inform interventions targeting early childhood nutrition, parental education, and targeted community to reduce persistent health disparities across Eastern Africa.

## Figures and Tables

**Table 1:** Descriptive statistics of potential covariates associated with child undernutrition in Eastern African countries (n = 37,570).

Attributes	Categories	Ethiopia (EDHS-2019)	Kenya (KDHS-2022)	Madagascar (MDHS-2021)	Tanzania (TDHS-2022)
		Frequency (%)	Frequency (%)	Frequency (%)	Frequency (%)
Socio-Demographic Characteristics					
Place of residence	Urban	1328 (23.08)	6686 (34.23)	2387 (19.10)	2938 (27.25)
	Rural	4425 (76.92)	12,844 (65.77)	10,112 (80.90)	7845 (72.75)
Source of drinking water	Unimproved	4797 (83.38)	13,803 (70.68)	10,067 (80.54)	8672 (80.42)
	Improved	956 (16.62)	5727 (29.32)	2432 (19.46)	2111 (19.58)
Toilet facility	Unimproved	4589 (79.77)	8245 (42.22)	9402 (75.22)	3486 (32.33)
	Improved	1164 (20.23)	11,285 (57.78)	3097 (24.78)	7297 (67.67)
Socio-economic status	Poor	2958 (51.42)	9762 (49.98)	6263 (50.11)	4416 (40.95)
	Middle	805 (13.99)	3379 (17.30)	2368 (18.95)	2222 (20.61)
	Rich	1990 (34.59)	6389 (32.71)	3868 (30.95)	4145 (38.44)
Father education	No education	-	3566 (22.37)	2726 (27.19)	1442 (15.98)
	Primary	-	5420 (34.00)	4301 (42.91)	5124 (56.78)
	Secondary	-	4145 (17.63)	2623 (3.73)	2205 (2.80)
	Higher	-	2810 (26.00)	374 (26.17)	253 (24.43)
**Child Characteristics**					
Milk consumption	No	2479 (73.56)	3104 (52.80)	6697 (91.98)	6184 (92.78)
	Yes	891 (26.44)	2775 (47.20)	584 (8.02)	481 (7.22)
Initiation of breastfeeding	Immediately	2988 (88.40)	5044 (79.22)	7403 (79.13)	5740 (80.21)
	Within first hour	361 (10.68)	1198 (18.82)	1453 (15.53)	1359 (19.00)
	Within first day	31 (0.92)	125 (1.96)	500 (5.34)	52 (0.73)
Birth order number	1st born	1261 (21.9)	5161 (26.4)	3453 (27.63)	2413 (22.38)
	2nd–4th	2598 (45.2)	9753 (49.9)	6113 (48.91)	5421 (50.27)
	>5	1894 (32.9)	4616 (23.6)	2933 (23.47)	2949 (27.35)
Sex of child	Male	2969 (51.61)	9950 (50.95)	6362 (50.90)	5489 (50.90)
	Female	2784 (48.39)	9580 (49.05)	6137 (49.10)	5294 (49.10)
Size of child at birth	Small	-	891 (14.49)	4089 (32.71)	1904 (28.96)
	Average	-	4105 (66.77)	5301 (42.41)	3820 (58.11)
	Large	-	1152 (18.74)	3109 (24.87)	850 (12.93)
Baby postnatal checkup within 2 months	No	3438 (86.60)	7602 (73.03)	6194 (66.81)	1591 (33.52)
	Yes	532 (13.40)	2807 (26.97)	3077 (33.19)	3155 (66.48)
Child’s age in months	0–6	688 (13.03)	2297 (12.87)	797 (13.44)	654 (13.25)
	7–12	507 (9.60)	2020 (11.32)	686 (11.57)	524 (10.62)
	13–24	1034 (19.58)	3542 (19.85)	1203 (20.29)	1065 (21.58)
	25–36	1058 (20.04)	3464 (19.41)	1154 (19.46)	953 (19.31)
	37–48	1034 (19.58)	3493 (19.57)	1123 (18.94)	925 (18.74)
	49–60	959 (18.16)	3031 (16.98)	966 (16.29)	814 (16.49)
**Maternal Characteristics**					
Breastfed ever	No	1750 (33.71)	9219 (47.20)	5293 (42.35)	5159 (47.84)
	Yes	3441 (66.29)	10,311 (52.80)	7206 (57.65)	5624 (52.16)
Maternal smoking	No	-	10,198 (99.32)	12,383 (99.07)	10,743 (99.63)
	Yes	-	70 (0.68)	116 (0.93)	40 (0.37)
Intake of iron during pregnancy	No	1672 (42.02)	1080 (10.37)	2546 (27.33)	1035 (17.77)
	Yes	2307 (57.98)	9332 (89.63)	6769 (72.67)	4790 (82.23)
Mother education	No education	3149 (54.74)	4464 (22.86)	3169 (25.35)	2274 (21.09)
	Primary	1823 (31.69)	6896 (35.31)	5468 (43.75)	5621 (52.13)
	Secondary	480 (8.34)	5542 (28.38)	3557 (28.46)	2775 (25.73)
	Higher	301 (5.23)	2628 (13.46)	305 (2.44)	113 (1.05)
Mother occupation	Not working	-	9657 (49.49)	1661 (13.29)	3641 (33.77)
	Working	-	9858 (50.51)	10,838 (86.71)	7142 (66.23)

**Table 2:** Response variable (CIAF) construction using different groups.

Groups	Description	Stunting (HAZ)	Wasting (WHZ)	Underweight (WAZ)	Ethiopia (EDHS-2019)	Kenya (KDHS-2022)	Madagascar (MDHS-2021)	Tanzania (TDHS-2022)
					N (%)	N (%)	N (%)	N (%)
**A**	No Failure	No	No	No	3049 (57.75)	13,457 (75.58)	3295 (55.57)	4450 (67.73)
**Anthropometric failure group**								
**B**	Wasting only	No	Yes	No	141 (2.67)	446 (2.50)	113 (1.91)	24 (6.49)
**C**	Wasting and Underweight	No	Yes	Yes	163 (3.09)	485 (2.72)	149 (2.51)	11 (6.22)
**D**	Wasting, Stunting, and Underweight	Yes	Yes	Yes	181 (3.43)	337 (1.89)	187 (3.15)	3 (1.60)
**E**	Stunting and Underweight	Yes	No	Yes	776 (14.70)	1137 (6.39)	928 (15.65)	79 (3.00)
**F**	Stunting only	Yes	No	No	868 (16.44)	1677 (9.42)	1142 (19.26)	323 (7.98)
**Y**	Underweight only	No	No	Yes	102 (1.93)	267 (1.50)	115 (1.94)	41 (6.97)

**Table 3:** Anthropometric failure (CIAF)-based percentage distribution of socio-demographic, child, and maternal characteristics.

Attributes	Types	Ethiopia (EDHS-2019) CIAF n (%)		Kenya (KDHS-2022) CIAF n (%)		Madagascar (MDHS-2021) CIAF n (%)		Tanzania (TDHS-2022) CIAF n (%)	
		Yes	No	Yes	No	Yes	No	Yes	No
Socio-Demographic Characteristics									
**Place of residence**	Urban	355 (6.72)	861 (16.31)	1068 (6.00)	4821 (27.08)	466 (7.86)	650 (10.96)	332 (6.73)	992 (20.12)
	Rural	1876 (35.53)	2188 (41.44)	3281 (18.43)	8636 (48.50)	2168 (36.57)	2645 (44.61)	1259 (25.53)	2348 (47.62)
**Source of drinking water**	Unimproved	1840 (34.85)	2567 (48.62)	3117 (17.51)	9387 (52.72)	2141 (36.11)	2665 (44.95)	1281 (25.98)	2721 (55.18)
	Improved	391 (7.41)	482 (9.13)	1232 (6.92)	4070 (22.86)	493 (8.32)	630 (10.63)	310 (6.29)	619 (12.55)
**Toilet facility**	Unimproved	1891 (35.81)	2318 (43.90)	2380 (13.37)	5208 (29.25)	1998 (33.70)	2456 (41.42)	566 (11.48)	993 (20.14)
	Improved	340 (6.44)	731 (13.84)	1969 (11.06)	8249 (46.33)	636 (10.73)	839 (14.15)	1025 (20.79)	2347 (47.60)
**Socio-economic status**	Poor	1329 (25.17)	1380 (26.14)	2926 (16.43)	6166 (34.63)	1400 (23.61)	1519 (25.62)	796 (16.14)	1244 (25.23)
	Middle	326 (6.17)	412 (7.80)	644 (3.62)	2435 (13.68)	510 (8.60)	668 (11.27)	343 (6.96)	675 (13.69)
	Rich	576 (10.91)	1257 (23.81)	779 (4.37)	4856 (27.27)	724 (12.21)	1108 (18.69)	452 (9.17)	1421 (28.82)
**Father education**	No education	-	-	1211 (8.15)	2133 (14.35)	589 (12.22)	657 (13.64)	243 (5.79)	480 (11.44)
	Primary	-	-	1314 (8.84)	3738 (25.16)	974 (20.22)	1123 (23.31)	837 (19.96)	1560 (37.20)
	Secondary	-	-	745 (5.01)	3083 (20.75)	517 (10.73)	772 (16.02)	249 (5.94)	714 (17.02)
	Higher	-	-	345 (2.32)	2290 (15.41)	47 (0.98)	139 (2.89)	17 (0.41)	94 (2.24)
**Child Characteristics**									
**Milk consumption**	No	976 (30.10)	1417 (43.71)	705 (12.54)	2255 (40.10)	1445 (40.24)	1845 (51.38)	953 (29.95)	2022 (63.54)
	Yes	291 (8.98)	558 (17.21)	707 (12.57)	1956 (34.79)	128 (3.56)	173 (4.82)	64 (2.01)	143 (4.49)
**Initiation of breastfeed**	Immediately	1191 (36.61)	1687 (51.86)	1212 (20.39)	3492 (58.76)	1581 (35.41)	1933 (43.29)	886 (26.22)	1817 (53.77)
	Within first Hour	126 (3.87)	221 (6.79)	302 (5.08)	820 (13.80)	310 (6.94)	392 (8.78)	191 (5.65)	467 (13.82)
	Within first day	10 (0.31)	18 (0.55)	33 (0.56)	84 (1.41)	94 (2.11)	155 (3.47)	4 (0.12)	14 (0.41)
**Birth order number**	1st born	416 (7.88)	694 (13.14)	909 (5.11)	3546 (19.91)	665 (11.22)	912 (15.38)	364 (7.38)	701 (14.22)
	2nd-4th	1010 (19.13)	1420 (26.89)	2074 (11.65)	6963 (39.10)	1257 (21.20)	1649 (27.81)	758 (15.37)	1755 (35.59)
	>5	805 (15.25)	935 (17.71)	1366 (7.67)	2948 (16.56)	712 (12.01)	734 (12.38)	469 (9.51)	884 (17.93)
**Sex of child**	Male	1201 (22.75)	1503 (28.47)	2411 (13.54)	6646 (37.32)	1436 (24.22)	1549 (26.13)	876 (17.77)	1601 (32.47)
	Female	1030 (19.51)	1546 (29.28)	1938 (10.88)	6811 (38.25)	1198 (20.21)	1746 (29.45)	715 (14.50)	1739 (35.27)
**Size of child at birth**	Small	-	-	128 (2.23)	700 (12.22)	728 (12.28)	1227 (20.69)	276 (8.85)	639 (20.50)
	Average	-	-	922 (16.10)	2935 (51.24)	1130 (19.06)	1413 (23.83)	580 (18.61)	1245 (39.94)
	Large	-	-	384 (6.70)	659 (11.50)	776 (13.09)	655 (11.05)	172 (5.52)	205 (6.58)
**Baby postnatal checkup within 2 months**	No	1299 (34.98)	1911 (51.45)	1674 (17.15)	5435 (55.67)	1312 (28.98)	1732 (38.25)	254 (11.03)	552 (23.97)
	Yes	199 (5.36)	305 (8.21)	642 (6.58)	2012 (20.61)	620 (13.69)	864 (19.08)	462 (20.06)	1035 (44.94)
**Child’s age in months**	0–6	186 (3.52)	502 (9.51)	364 (2.04)	1932 (10.85)	251 (4.23)	546 (9.21)	141 (2.86)	513 (10.40)
	7–12	154 (2.92)	353 (6.69)	441 (2.48)	1578 (8.86)	256 (4.32)	430 (7.25)	141 (2.86)	383 (7.77)
	13–24	449 (8.50)	585 (11.08)	1029 (5.78)	2513 (14.11)	607 (10.24)	596 (10.05)	409 (8.29)	655 (13.28)
	25–36	519 (9.83)	539 (10.21)	965 (5.42)	2495 (14.01)	564 (9.51)	590 (9.95)	358 (7.26)	595 (12.07)
	37–48	473 (8.96)	561 (10.63)	867 (4.87)	2624 (14.74)	544 (9.18)	579 (9.77)	309 (6.27)	615 (12.47)
	49–60	450 (8.52)	509 (9.64)	683 (3.84)	2315 (13.00)	412 (6.95)	554 (9.34)	233 (4.73)	579 (11.74)
**Maternal Characteristics**									
**Breastfed ever**	No	659 (13.81)	814 (17.05)	1860 (10.45)	6054 (34.00)	1065 (17.96)	1337 (22.55)	775 (15.72)	1527 (30.97)
	Yes	1360 (28.49)	1940 (40.65)	2489 (13.98)	7403 (41.58)	1569 (26.46)	1958 (33.02)	816 (16.55)	1813 (36.77)
**Maternal smoking**	No	-	-	2301 (24.59)	6993 (74.73)	2615 (44.11)	3273 (55.20)	1586 (32.16)	3327 (67.47)
	Yes	-	-	22 (0.24)	42 (0.45)	19 (0.32)	22 (0.37)	5 (0.10)	13 (0.26)
**Intake of iron during pregnancy**	No	648 (17.41)	887 (23.84)	296 (3.02)	724 (7.38)	558 (12.26)	696 (15.30)	179 (6.37)	343 (12.20)
	Yes	853 (22.92)	1333 (35.82)	2032 (20.72)	6757 (68.89)	1383 (30.40)	1913 (42.04)	707 (25.14)	1583 (56.29)
**Mother education**	No education	1395 (26.42)	1516 (28.71)	1473 (8.27)	2691 (15.11)	676 (11.40)	843 (14.22)	408 (8.27)	670 (13.59)
	Primary	670 (12.69)	972 (18.41)	1632 (9.17)	4613 (25.91)	1217 (20.53)	1326 (22.36)	869 (17.62)	1710 (34.68)
	Secondary	111 (2.10)	333 (6.31)	956 (5.37)	4035 (22.66)	691 (11.65)	1007 (16.98)	310 (6.29)	916 (18.58)
	Higher	55 (1.04)	228 (4.32)	288 (1.62)	2118 (11.89)	50 (0.84)	119 (2.01)	4 (0.08)	44 (0.89)
**Mother occupation**	Not working	-	-	2398 (13.48)	6545 (36.79)	312 (5.26)	455 (7.67)	536 (10.87)	1190 (24.13)
	Working	-	-	1947 (10.94)	6902 (38.79)	2322 (39.16)	2840 (47.90)	1055 (21.40)	2150 (43.60)

**Table 4:** Multivariable logistic regression analysis of the CIAF-based factors associated with undernutrition in children of five and under.

Attributes	Categories	Anthropometric Failure Using the CIAF			
		Ethiopia (EDHS-2019)	Kenya (KDHS-2022)	Madagascar (MDHS-2021)	Tanzania (TDHS-2022)
		AOR (*p*-Value) (C.I)	AOR (*p*-Value) (C.I)	AOR (*p*-Value) (C.I)	AOR (*p*-Value) (C.I)
Socio-Demographic Characteristics					
**Place of residence**	Urban *	-	-	-	-
	Rural	1.010 (0.952) (0.721–1.414)	1.064 (0.681) (0.790–1.432)	1.230 (0.239) (0.871–1.737)	1.305 (0.164) (0.896–1.902)
**Source of drinking water**	Unimproved *	-	-	-	-
	Improved	1.128 (0.413) (0.845–1.506)	0.842 (0.128) (0.676–1.050)	0.997 (0.987) (0.745–1.335)	0.983 (0.924) (0.697–1.386)
**Toilet facility**	Unimproved *	-	-	-	-
	Improved	0.829 (0.259) (0.600–1.146)	0.878 (0.280) (0.693–1.111)	1.053 (0.719) (0.794–1.396)	1.060 (0.728) (0.760–1.478)
**Socio-economic status**	Poor *	-	-	-	-
	Middle	0.940 (0.703) (0.685–1.289)	0.683 **(0.021)** (0.494–0.944)	0.822 (0.226) (0.598–1.129)	1.046 (0.814) (0.715–1.531)
	Rich	0.645 **(0.005)** (0.475–0.875)	0.535 **(0.001)** (0.367–0.780)	0.988 (0.948) (0.689–1.415)	0.871 (0.520) (0.572–1.325)
**Father education**	No education *	-	-	-	-
	Primary	-	0.974 (0.879) (0.695–1.363)	0.747 (0.053) (0.557–1.004)	1.297 (0.213) (0.861–1.956)
	Secondary	-	0.589 **(0.009)** (0.397–0.873)	0.631 **(0.018)** (0.432–0.923)	1.240 (0.399) (0.751–2.048)
	Higher	-	0.436 **(0.001)** (0.264–0.720)	0.369 **(0.019)** (0.161–0.847)	0.700 (0.548) (0.219–2.235)
**Child characteristics**					
**Milk consumption**	No *	-	-	-	-
	Yes	0.762 (0.645) (0.585–0.994)	1.092 (0.417) (0.882–1.353)	1.055 (0.789) (0.709–1.570)	1.132 (0.655) (0.655–1.954)
**Initiation of breastfeed**	Immediately *	-	-	-	-
	Within first hour	0.869 (0.428) (0.614–1.229)	1.021 (0.867) (0.794–1.314)	0.957 (0.782) (0.705–1.299)	0.796 (0.233) (0.547–1.158)
	Within first day	1.614 (0.415) (0.509–5.112)	0.936 (0.866) (0.438–2.002)	0.700 (0.178) (0.417–1.175)	1.37 (0.983) (0.675–2.789)
**Birth order number**	1st born *	-	-	-	-
	2nd–4th	1.077 (0.665) (0.769–1.506)	1.023 (0.888) (0.745–1.404)	0.765 (0.140) (0.536–1.091)	0.607 (0.431) (0.386–1.355)
	>5	1.045 (0.843) (0.672–1.625)	1.233 (0.342) (0.800–1.899)	0.733 (0.228) (0.443–1.213)	0.760191 (0.374) (0.415–1.390)
**Sex of child**	Male *	-	-	-	-
	Female	0.759 **(0.012)** (0.613–0.941)	0.569 **(<0.0001)** (0.465–0.696)	0.477 **(<0.0001)** (0.379–0.599)	0.692 **(0.008)** (0.528–0.907)
**Size of child at birth**	Small *	-	-	-	-
	Average	-	0.645 (0.204) (0.172–1.309)	1.190 (0.190) (0.717–1.543)	0.981 (0.902) (0.726–1.325)
	Large	-	0.777 (0.147) (0.585–1.990)	0.351 (0.258) (0.232–1.590)	0.345 (0.439) (0.199–1.535)
**Baby postnatal checkup within 2 months**	No *	-	-	-	-
	Yes	0.973 (0.877) (0.691–1.370)	1.010 (0.930) (0.801–1.273)	0.309 **(0.032)** (0.24–0.673)	1.117 (0.448) (0.838–1.489)
**Child’s age in months**	0–6 *	-	-	-	-
	7–12	1.391 **(0.030)** (1.033–1.873)	1.692 **(<0.001)** (1.273–2.250)	1.296 (0.087) (0.962–1.746)	1.286 (0.199) (0.876–1.888)
	13–24	2.642 **(<0.001)** (2.024–3.449)	2.373 **(<0.001)** (1.790–3.144)	2.083 **(<0.001)** (1.571–2.761)	1.930 **(<0.001)** (1.370–2.720)
	25–36	0.265 (0.168) (0.135–1.538)	0.786 (0.556) (0.234–1.457)	0.536 (0.432) (0.236–1.454)	0.235 (0.432) (0.155–1.544)
	37–48	0.235 (0.887) (0.17–2.876)	0.567 (0.876) (0.347–2.588)	0.256 (0.211) (0.167–2.466)	0.667 (0.453) (0.235–1.554)
	49–60	1.219 (0.467) (1.154–2.546)	1.656 (0.121) (1.444–1.998)	1.313 (0.674) (1.247–2.358)	1.458 (0.654) (1.157–2.468)
**Maternal characteristics**					
**Breastfed ever**	No *	-	-	-	-
	Yes	1.207 (0.290) (0.851–1.711)	1.110 (0.501) (0.818–1.504)	0.894 (0.572) (0.607–1.317)	0.754 (0.128) (0.525–1.084)
**Maternal smoking**	No *	-	-	-	-
	Yes	-	0.661 (0.436) (0.233–1.871)	0.468 (0.364) (0.21–1.410)	1.35 (0.445) (0.896–2.342)
**Intake of iron during pregnancy**	No *	-	-	-	-
	Yes	1.126 (0.307) (0.895–1.417)	1.076 (0.646) (0.786–1.473)	0.848 (0.219) (0.653–1.102)	0.917 (0.637) (0.641–1.312)
**Mother education**	No education *	-	-	-	-
	Primary	0.794 (0.086) (0.610–1.032)	0.899 (0.540) (0.640–1.262)	1.309 (0.088) (0.960–1.784)	0.654 **(0.025)** (0.451–0.949)
	Secondary	0.547 **(0.007)** (0.353–0.848)	1.156 (0.481) (0.771–1.732)	1.017 (0.934) (0.680–1.519)	0.606 **(0.039)** (0.377–0.974)
	Higher	0.365 **(0.001)** (0.201–0.664)	1.184 (0.547) (0.682–2.056)	0.732 (0.548) (0.265–2.023)	0.324 (0.346) (0.157–1.459)
**Mother occupation**	Not working *	-	-	-	-
	Working	-	1.135 (0.267) (0.907–1.421)	1.031 (0.847) (0.755–1.407)	1.111 (0.462) (0.839–1.471)

**Note:** * shows the reference category; Bold values indicate statistically significant *p*-values (*p* < 0.05); C.I: Confidence Interval, AOR: Adjusted Odds Ratio.

**Table 5:** The diagnostic test summary for assessing the goodness-of-fit of anthropometric failure (CIAF) models.

	Models			
Tests	Ethiopia (EDHS-2019)	Kenya (KDHS-2022)	Madagascar (MDHS-2021)	Tanzania (TDHS-2022)
	χ^2^ (*p*-Values)	χ^2^ (*p*-Values)	χ^2^ (*p*-Values)	χ^2^ (*p*-Values)
Pearson-Chi Square test	123.52 (0.419)	199.72 (0.355)	101.75 (0.104)	46.86 (0.153)
Hosmer–Lemeshow test	4.24 (0.835)	13.92 (0.287)	3.57 (0.893)	12.11 (0.446)

## Data Availability

The data set used in the study taken from the Demographic and Health Survey (DHS) website and the files are available at the following url: All data files are available accessed on 4 July 2025 at the following url: https://dhsprogram.com/data/dataset/Madagascar_Standard-DHS_2021.cfm?flag=1; https://dhsprogram.com/data/dataset/Kenya_Standard-DHS_2022.cfm?flag=1; https://dhsprogram.com/data/dataset/Tanzania_Standard-DHS_2022.cfm?flag=1.
